# Fatigue and Suicidal Ideation in People With Multiple Sclerosis: The Role of Social Support

**DOI:** 10.3389/fpsyg.2020.00504

**Published:** 2020-03-18

**Authors:** Pavol Mikula, Vladimira Timkova, Marcela Linkova, Marianna Vitkova, Jarmila Szilasiova, Iveta Nagyova

**Affiliations:** ^1^Department of Social and Behavioural Medicine, Faculty of Medicine, Pavol Jozef Safarik University in Košice, Košice, Slovakia; ^2^Department of Neurology, Faculty of Medicine, Pavol Jozef Safarik University in Košice, Košice, Slovakia

**Keywords:** suicidal ideation, social support, fatigue, sleep quality, multiple sclerosis

## Abstract

Fatigue and poor sleep quality are among the most common patient-reported problems associated with multiple sclerosis (MS). Social support, on the other hand, is often found to be positively associated with quality of life in patients with neurological diseases. Studies also show that suicidal ideation (SI) levels in MS are elevated compared to the general population. Thus, the aim of this study is to assess the associations between fatigue, social support, and SI in patients with MS. Out of 184 MS patients asked to participate in this cross-sectional study, 156 agreed (RR 69.8%; 75% female; mean age: 39.95 ± 9.97 years). Patients filled-in the Multidimensional Fatigue Inventory-20, the Pittsburgh Sleep Quality Index, the Multidimensional Scale of Perceived Social Support and the subscale of the General Health Questionnaire-28 focused on assessing SI. Models were controlled for age, gender, disease duration, functional disability, and sleep quality. Data were analyzed using multiple linear regressions. SI was positively associated with lower sleep quality and four types of fatigue: general, mental, reduced activity, and reduced motivation (*p* < 0.05). Physical fatigue was not significantly associated with SI. Social support was negatively associated with SI in all models. The final models under study explained from 24.3 to 29.7% of the total variance in SI. SI yielded associations with both sleep quality and fatigue, with the exception of physical fatigue. Information provided by physicians on sleep management, and a psychosocial intervention focused on people who provide support for patients with MS (family, friends, and significant others) may reduce levels of SI.

## Introduction

Multiple sclerosis (MS) is a chronic neurological disease affecting the central nervous system (CNS) by inflammatory processes, the breakdown of the myelin, and consequently damage to the axons (Compston and Coles, 2002). The changes occurring in the CNS vary from patient to patient, and while the symptomatology is usually patient-specific and variable for different people, there are four recognized MS courses: clinically isolated syndrome (CIS), relapsing-remitting MS (RRMS), primary progressive MS (PPMS), and secondary progressive MS (SPMS) (Lublin et al., 2014). Symptomatology of MS include visual problems, balance problems, vertigo, paresis, sensory disturbances, sphincter problems, spasticity, pain, and fatigue in addition to other manifestations (Barin et al., 2018).

The majority of MS patients consider fatigue as one of their most severe symptoms, and chronic fatigue affects many daily activities and thus lowers their physical, mental, and social quality of life (Brassington and Marsh, 1998; Wilcon et al., 2011; Cehelyk et al., 2019). Previous research shows that fatigue may also be associated with depressive symptomatology and inactivity, which consequently leads to other negative psychological correlates (Mileic et al., 2011). Fatigue seems to be subjectively perceived as a symptom affecting or being associated with other MS symptoms, such as pain and movement problems. High level of perceived fatigue was found to be significantly associated with poorer self-reported sleep quality (Al-Dughmi and Siengsukon, 2016), although this particular connection between sleep quality and fatigue in MS patients has not been studied in great detail, to our knowledge. Poor sleep quality was nonetheless found to be a symptom of importance which leads to greater disability and problems in performing of daily activities for MS patients (Vitkova et al., 2018; Newland et al., 2019). Furthermore, some previous research has shown that sleep quality may be a useful indicator for SI risk (Kim et al., 2013).

Among the symptoms of a more psychological nature is cognitive impairment or increased prevalence of depression (Barry et al., 2018). Depressive symptomatology is often associated with suicidal ideation (SI) in people with MS (Tauil et al., 2018). Suicidal thoughts, preoccupation with suicide, or even suicidal attempts are more prevalent in people suffering from MS as well as in a number of other neurological disorders, such as Huntington’s disease, Alzheimer’s disease, amyotrophic lateral sclerosis, idiopathic neuropathy or epilepsy, compared to the general population (Lewis et al., 2017; Eliasen et al., 2018). The reason for the higher prevalence of SI in a number of chronic diseases, including MS, may be that SI may offer some patients a mechanism for feeling in control of their lives in the face of a daunting, unpredictable disease (Gaskill et al., 2011). Another reason for depression going undiagnosed and SI not reported may be the effort of MS patients to minimize these problems during medical assessment in order to reassure clinicians that they will not harm themselves (Caine and Schwid, 2002). Another explanation may be rooted in more attention being focused on the treatment of physical correlates of the diseases and less on psychological symptoms, as medical professionals have often too little time or expertise to assess the psychological health of patients (Østbye et al., 2005). As a result, many psychiatric comorbidities go untreated and unrecognized (Rickards, 2006).

This lack of time focused on psychiatric comorbidities in formal primary care can be mitigated by informal social support, which refers to emotional, instrumental, and informational support that people receive from support providers (family members, friends, or significant others) in order to cope with various stressful events in their life (Bruchon-Schweitzer and Boujut, 2014). Different forms of social support from family and friends seem to diminish the negative effects of some symptoms of chronic conditions, such as dementia, fibromyalgia, rheumatoid arthritis, or osteoarthritis (Kruithof et al., 2013; Eshkoor et al., 2014). Literature concerning MS shows a positive effect of social support on various mental health concepts and symptoms, like anxiety or depression (Henry et al., 2019). On the other hand, increased family tension, loneliness, and social isolation were found to be significantly associated with SI in patients suffering from various chronic conditions (Rückert-Eheberg et al., 2019), including MS (Feinstein, 2002; Gaskill et al., 2011).

Despite the recognition of an elevated suicide risk in people with MS, very little is known about associated risk factors, including sleep-related problems and the role of social support. To our best knowledge, no previous studies have addressed the associations between sleep related problems, social support, and SI in MS patients so far. Thus, the aim of this study is to assess associations between sleep quality, fatigue, social support, and SI in patients with MS, when controlled for age, gender, disease duration, and functional disability. Based on results of the abovementioned studies (e.g., Feinstein, 2002; Gaskill et al., 2011; Mileic et al., 2011; Kim et al., 2013; Henry et al., 2019; Isernia et al., 2019) we hypothesized (1) the presence of the positive associations between sleep quality, fatigue, and SI in patients with MS and (2) the presence of negative association between social support and SI in patients with MS.

## Methods

### Participants and Procedure

Out of 184 patients asked to participate in the study, 156 agreed (response rate: 84.7%, 75% females, mean age 39.95 ± 97 years). After signing the informed consent, the patients filled in the questionnaires and underwent a neurological examination. All the patients included in the study met the McDonald MS diagnostic criteria (Polman et al., 2011). Exclusion criteria were pregnancy, inability to speak Slovak and cognitive dysfunction determined by the Mini Mental State Examination (score of <24). None of the participants were excluded due to these criteria. The data collection took place at the Department of Neurology of the University Hospital of Louis Pasteur in Kosice and patients who were asked to participate in the study were diagnosed MS patients who regularly visit the neurological outpatient clinic. The procedure started by sending out an invitation, an informed consent letter, questionnaires, and a non-response sheet to the patients via postal mail. After 2 weeks, a trained interviewer called each patient in order to arrange a face-to-face interview, allowing for clarification of the patient’s responses and answering their inquiries. A neurological examination was performed right after the interview, with no personal identifiers being included in the database. The local ethics committee approved the study. All participants were provided with information about the study and signed an informed consent statement prior to the study.

### Measures

Questionnaires used in this study underwent translation and back-translation from the original language into Slovak and back again by certified translators in order to ensure that the meaning of the items were not lost in translation.

#### Suicidal Ideation

This variable was measured using a subscale of the 28-item General Health Questionnaire (GHQ-28) (Goldberg and Hillier, 1979). Out of 28 items, 4 directly address SI: “Have you thought about the possibility of killing yourself?”, “Do you wish to be dead and far away from everything?”, “Do you have continual thoughts about ending your life?”, and “Do you have the impression that life is not worth of living?” These items asked participants if they experienced such occurrences in past 4 weeks and each of them was scored on a 4-point Likert scale, with a higher score indicating a higher prevalence of SI. The score thus ranges from 4 to 16 (Goldberg et al., 1998). This study treats SI as a continuous variable. The Cronbach’s alpha for the four SI items in our sample was 0.88.

#### Fatigue

The Multidimensional Fatigue Inventory-20 (MFI-20) was used to determine the levels of fatigue among people with MS (Smets et al., 1995). It consists of five subscales – general fatigue, physical fatigue, reduced activity, reduced motivation, and mental fatigue – and each subscale has four items. People with MS were given statements such as: “I feel very active,” or “Physically I feel only able to do a little”. The answers on a five-point Likert scale were anchored in quotes: “Yes, that is true” and “No, that is not true”. Some of the items were worded in a negative way and had to be recoded prior to the statistical analyses. A higher final score in each of the subscales indicated more prevalent fatigue and a lower score less prevalent fatigue. Cronbach’s alpha in our sample was 0.79 for general fatigue, 0.84 for physical fatigue, 0.82 for reduced activity, 0.64 for reduced motivation, and 0.78 for mental fatigue.

#### Sleep Quality

Sleep quality was measured using The Pittsburgh Sleep Quality Index (PSQI). This questionnaire consists of 19 items, which generate 7 subscales (subjective sleep quality, sleep latency, sleep duration, habitual sleep efficiency, sleep disturbances, use of sleep medication, and daytime dysfunction). The PSQI also provides a global score for all the subscales. This score ranges from 0 to 21, with a higher number indicating worse sleep quality (Buysse et al., 1989). The Cronbach’s alpha in our sample was 0.76.

#### Social Support

Social support was measured using the Multidimensional Scale of Perceived Social Support (MSPSS), which is used to assess subjective perception of social support from a significant other, family and friends, as well as total social support from all these sources (Zimet et al., 1988). The MSPSS is a 12-item questionnaire with 7-point Likert scale, with responses to the questions ranging from 1 to 7. The higher number indicates higher perceived social support in all of the items. The score ranges from 12 to 84. Cronbach’s alpha for the whole scale was 0.95.

#### Clinical Variables

The Kurtzke Expanded Disability Status Scale (EDSS) was used to assess the functional disability of people with MS. The score in this scale ranges from 0.0 to 10.0, with higher score indicating more severe disability (Kurtzke, 1983). EDSS was measured by a neurologist during a neurological examination. Data on the disease duration and course of MS were provided by the neurologist as well. Basic sociodemographic data were collected during an interview with a trained interviewer.

#### HADS

To control for the role of depression in our model, we used Hospital Anxiety and Depression Scale (HADS) (Zigmond and Snaith, 1983). The scale consists of 14 items, 7 of which are related to depression and 7 to anxiety. The four-point scale is scored as a Likert scale. The score ranges from 0 to 21, with higher score representing more distress in each scale.

### Statistical Analyses

First, descriptive analyses of the sample were carried out. Next, the bivariate correlations between the independent variables of our models (sleep quality, types of fatigue, social support, and EDSS) and the dependent variable (SI) were calculated. We used partial correlations to control the effect of depression (measured by HADS) (Zigmond and Snaith, 1983) on the associations between variables under study. Finally, linear regression analyses were performed with SI as the dependent variable. The analyses were performed in the IBM SPSS 23 software.

## Results

Table 1 displays the description of the study population (*N* = 156). The sample averaged 39.95 ± 9.97 years old and consisted of 75% women. The majority of the sample (72.4%) had a secondary education. The mean score for functional disability, as measured by the EDSS, was 3.10 ± 1.37 and for disease duration it was 7.40 ± 5.48. The percentage of MS patients with SI was 14.10%.

**TABLE 1:** Description of the study population.

Variables	*N* (%)	Mean	Range
Age	156	39.959.97	18–61
**Gender**			
Male	39 (25%)		
Female	117 (75%)		
**Education**			
Elementary	6 (3.8%)		
Secondary	113 (72.4%)		
University	37 (23.8%)		
Disease duration (years)	151	7.405.48	1–28
EDSS	141	3.101.37	1–8
**MS course**			
CIS	16 (11.5%)		
RRMS	97 (69.8%)		
SPMS	26 (18.7%)		
PSQI	155	5.773.54	0–16
MFI_GF	156	13.944.43	4–20
MFI_PF	156	13.374.86	4–20
MFI_RA	156	10.894.75	4–20
MFI_RM	156	8.523.64	4–20
MFI_MF	156	10.374.47	4–20
MSPSS	156	67.1013.56	12–84
GHQ_SI	156	5.822.59	4–14

*Missing data: Disease duration (3.2%); EDSS (9.6%); PSQI (0.6%); MS course (10.8%). EDSS, Expanded Disability Status Scale; CIS, clinically isolated syndrome; RRMS, relapsing remitting multiple sclerosis; SPMS, secondary progressive multiple sclerosis; PSQI, The Pittsburgh Sleep Quality Index; MFI_GF, general fatigue; MFI_PF, physical fatigue; MFI_RA, reduced activity; MFI_RM, reduced motivation; MFI_MF, mental fatigue; MSPSS, Multidimensional Scale of Perceived Social Support; GHQ_SI, suicidal ideation.*

Correlation analyses showed that sleep quality correlated moderately with different types of fatigue, while the correlation with social support was found to be negative. A similar occurrence was found in the case of social support and fatigue, as all five types showed slight to moderate levels of negative correlation. EDSS correlated with general fatigue (0.29), physical fatigue (0.41), and reduced activity (0.42) (Table 2). Controlling for depression (measured by HADS), showed that associations between variables under study and SI were significant, however, attenuated (Supplementary Material).

**TABLE 2:** Correlations between sleep quality, types of fatigue, social support, EDSS, and SI.

	Sleep quality	General fatigue	Physical fatigue	Reduced activity	Reduced motivation	Mental fatigue	Social support	EDSS
Sleep quality								
General fatigue	0.46*							
Physical fatigue	0.39*	0.80*						
Reduced activity	0.39*	0.67*	0.79*					
Reduced motivation	0.54*	0.58*	0.58*	0.66*				
Mental fatigue	0.48*	0.57*	0.43*	0.46*	0.63*			
Social support	−0.36*	−0.23*	−0.24*	−0.21*	−0.30*	−0.35*		
EDSS	0.04	0.29*	0.41*	0.42*	0.10	0.02	**−0.03**	
Suicidal ideation	0.44*	0.32*	0.25*	0.27*	0.43*	0.45*	−0.34*	−0.06

*EDSS, Expanded Disability Status Scale; ^∗^bold values: p < 0.05.*

Linear regression analyses (Table 3) were then performed. SI, as measured by the GHQ-28, was regressed on the basic sociodemographic variables (age, gender), objective medical assessment and medical history (EDSS, disease duration), five types of fatigue measured by the MFI-20 (general fatigue, physical fatigue, reduced activity, reduced motivation, and mental fatigue), sleep quality and social support. The mental fatigue model explained the most variance of SI from all the models under study (29.7%), and the physical fatigue model the least (24.3%). But all five models did significantly contribute to the explained variance of SI in people with MS. Sleep quality and social support were significant predictors in all models, and all but one type of fatigue (physical fatigue) were significant predictors, as well.

**TABLE 3:** Linear regression analyses: Suicidal ideation regressed on demographic variables, EDSS, disease duration, sleep quality, social support, and five types of fatigue.

	Variables	Beta	*F*	Adjusted *R*^2^
Model 1	Age	−0.12	7.924	0.260
	Gender	−0.01		
	EDSS	−0.01		
	Disease duration	−0.14		
	Sleep quality	0.27*		
	General fatigue	0.20*		
	Social support	−0.25*		
Model 2	Age	−0.12	7.329	0.243
	Gender	−0.00		
	EDSS	−0.02		
	Disease duration	−0.14		
	Sleep quality	0.31*		
	Physical fatigue	0.14		
	Social support	−0.25*		
Model 3	Age	−0.13	8.083	0.264
	Gender	−0.03		
	EDSS	−0.05		
	Disease duration	−0.12		
	Sleep quality	0.28*		
	Reduced activity	0.23*		
	Social support	−0.23*		
Model 4	Age	−0.16	8.837	0.284
	Gender	−0.03		
	EDSS	0.03		
	Disease duration	−0.13		
	Sleep quality	0.22*		
	Reduced motivation	0.28*		
	Social support	−0.22*		
Model 5	Age	−0.10	9.342	0.297
	Gender	−0.02		
	EDSS	0.03		
	Disease duration	−0.16		
	Sleep quality	0.24*		
	Mental fatigue	0.29*		
	Social support	−0.21*		

EDSS, Expanded Disability Status Scale; *bold values: *p* < 0.05.

## Discussion

The aim of this study was to assess associations between sleep quality, fatigue, social support, and SI in patients with MS, when controlled for age, gender, disease duration, and functional disability. We hypothesized (1) the presence of the positive associations between sleep quality, fatigue, and SI in patients with MS and (2) the presence of negative association between social support and SI in patients with MS. We found that SI, a potential harbinger of suicide, is more prevalent in our sample (14.1%) compared to the cross-national lifetime prevalence of SI (9.2%) in the general population and the annual prevalence (2.0–2.1%) of SI in the general population (Nock et al., 2008) and is significantly associated with poor sleep quality, fatigue, and lack of social support. Out of five models under consideration, four yielded similar results when it came to variables that significantly contributed to the explained variance of SI. Out of five types of fatigue, only physical fatigue did not significantly contribute to the explained variance in SI.

As SI and suicide-related behavior are associated with the presence of chronic disease (Joshi et al., 2017), it is important to recognize the risk factors as well as the protective factors that are able to mitigate the negative effects of chronic conditions on daily functioning and psychological impairment. Our study supports previous assumptions that SI may represent not only a symptom of major depression but may also be driven by neurobiological processes (Du et al., 2017), including sleep-related symptomatology.

We found no significant association between disability status and SI in MS patients. On the contrary, a recent study by Lewis et al. (2017) showed that different types of disability were associated with suicidality. A possible explanation for these discrepancies in results may stem from the different methodology utilized in data collection. To be more specific, Guy’s neurological disability scale, which was used in the research of Lewis et al. (2017), is oriented on self-reported disability assessment of patient, while the EDSS used in our study is assessed by a neurologist. This aforementioned difference in results may suggest that subjective perception of disability is more important in the development of SI compared to objective disability and could be utilized in changing disease perception in order to diminish SI in MS patients.

Furthermore, we found that physical fatigue was not significantly associated with SI in people with MS, while other types of fatigue, especially mental fatigue, were. Thus, it may be assumed, that SI may reflect mostly the mental, subjective correlates of fatigue and not objective correlates of functional disability, as measured by the EDSS and physical fatigue. This conclusion is supported by mental fatigue, which showed the strongest association with SI out of the five types of fatigue under study.

Social support also seems to be associated with SI, but unlike fatigue, the association seems to be a negative one. Our results confirm notions of the protective value of social support among many different age groups and populations both with and without chronic conditions (Sengul et al., 2014; Ariapooran and Khezeli, 2018; Fredrick et al., 2018). Our results suggest that social support may have a buffering effect, and that in case of a long-term health-threatening condition, social support from family, friends, and significant others may protect MS patients from more severe depressive and suicidal thoughts.

### Strengths and Limitations

To the best of our knowledge, this is the first study exploring the associations between sleep-related problems, social support, and SI in MS patients. Some limitations should be noted, however. First, no formal psychiatric diagnosis of SI was established; on the other hand, GHQ-28 items are frequently used to assess the presence of SI in various populations (Hamilton and Schweitzer, 2000; Gili-Planas et al., 2001; Watson et al., 2001; Kawabe et al., 2016). Furthermore, it should be noted that the four-item GHQ-28 SI subscale has shown good concurrent validity with Beck’s Suicide Intent Scale (Watson et al., 2001). Another limitation to be mentioned cross-sectional nature of the study, which does not allow for causal relationships to be drawn. Furthermore, after controlling for depression, the correlations between variables under study attenuated and thus it may be implied that depression may be possibly cofounding factor in the associations between SI and sleep problems, social support, and clinical variables. Final limitation concerns the lack of validity and reliability of HADS scale used to control for the role of depression (Coyne and van Sonderen, 2012).

### Implications for Practice and Future Research

Suicidal ideation is a potentially treatable cause of morbidity and mortality in MS. As SI may turn into suicide attempts, education of caregivers, and relatives about the occurrence of SI in patients with MS may be important and protective. Health care professionals should be educated in the detection of SI in MS patients at increased risk and refer them to relevant healthcare facilities, including psychiatric and psychological care. Because assessing suicidality in medical settings may be challenging, it may be beneficial to detect SI in neurological care with simple and economical screening tools, such as the GHQ-28. Physicians should develop communication skills, particularly relating to open and clear discussions of suicidality in MS patients, through various training courses (Kye and Park, 2017). Preventive strategies should include a close monitoring of pharmacotherapy to prevent progression of SI, awareness of caregivers about SI and early identification of psychosocial risk factors across a variety of healthcare and social instances the patient may encounter during their life with the disease (e.g., Mellentin et al., 2018).

Furthermore, our study identified that social support may be of importance in diminishing the level of SI in MS patients. Thus, adequate social support and its improvement by various educational strategies seems to be a protective factor in preventing SI in people with MS. Our study helped to identify the sleep-related variables linked with SI, which could be readily available from a routine clinical inquiry. Another implication of our study may lie in educating patients on the basic sleep hygiene, which may help to decrease poor sleep quality and fatigue and consequently to diminish the levels of SI, or at least mitigate its adverse effects. As SI may lead to suicidal behavior, suicide potential in MS patients should be assessed in future research. Moreover, it is important to identify the constituting risk factors to prevent suicidal behavior among vulnerable MS patients. Longitudinal studies are needed to confirm the associations revealed in our study. Finally, it would be interesting to compare the results of this study with other chronic conditions, especially of a neurological nature. As the association between SI and social support was no longer significant when controlled for depression, future studies should be conducted to identify protective factors against SI in depressed MS patients with MS. As the association between fatigue and SI attenuated when controlled for depression, future studies may shed some light using more valid and reliable measures of depression.

### Conclusion

The current study reveals three main points that deserve attention. First, SI rates in people with MS may be elevated compared to the general population due to disease-related symptoms. Second, our findings suggest that to reduce SI in patients with MS, efforts should focus on reducing poor sleep quality and fatigue. Third, negative effects of sleep-related problems on SI may be diminished by adequate social support. However, future longitudinal research is needed to confirm the causality.

## Data Availability Statement

The datasets are available on request. The raw data supporting the conclusions of this article will be made available by the authors, without undue reservation, to any qualified researcher.

## Ethics Statement

The studies involving human participants were reviewed and approved by the Local Ethics Committee of Faculty of Medicine of the Pavol Jozef Safarik University. The patients/participants provided their written informed consent to participate in this study.

## Author Contributions

PM, JS, and MV were responsible for data collection. PM and VT carried out the statistical analyses. All authors contributed to manuscript preparation and subsequent revisions.

## Conflict of Interest

The authors declare that the research was conducted in the absence of any commercial or financial relationships that could be construed as a potential conflict of interest.

## References

[B1] Al-Dughmi M. Siengsukon C. F. (2016). The relationship between sleep quality and perceived fatigue measured using the Neurological Fatigue Index in people with Multiple Sclerosis. *Neurol. Res.* 38 943–949. 10.1080/01616412.2016.1232014 27638459

[B2] Ariapooran S. Khezeli M. (2018). Suicidal ideation among divorced women in Kermanshah, Iran, the role of social support and psychological resilience. *Iran J. Psychiatr. Behav. Sci.* 12:e3565.

[B3] Barin L. Salmen A. Disanto G. Babačić H. Calabrese P. Chan A. (2018). The disease burden of multiple sclerosis from the individual and population perspective: which symptoms matter most? *Mult. Scler. Relat. Dis.* 25 112–121. 10.1016/j.msard.2018.07.013 30059895

[B4] Barry A. Cronin O. Ryan A. M. Sweeney B. O′Toole O. Allen A. P. (2018). Impact of short-term cycle ergometer training on quality of life, cognition and depressive symptomatology in multiple sclerosis patients: a pilot study. *Neurol. Sci.* 39 461–469. 10.1007/s10072-017-3230-0 29280019

[B5] Brassington J. S. Marsh N. V. (1998). Neuropsychological aspects of multiple sclerosis. *Neuropsychol. Rev.* 8 43–77. 9658410 10.1023/a:1025621700003

[B6] Bruchon-Schweitzer M. Boujut É. (2014). La personnalité comme facteur de vulnérabilité. *Psychol. Sup.* 195–276.

[B7] Buysse D. J. Reynolds C. F. Monk T. H. Berman S. R. Kupfer D. J. (1989). The Pittsburgh Sleep Quality Index: a new instrument for psychiatric practice and research. *Psychiatry Res.* 28 193–213. 10.1016/0165-1781(89)90047-4 2748771

[B8] Caine Schwid S. R. (2002). Multipe sclerosis, depression, and the risk of suicide. *Neurology* 59 662–663. 10.1212/wnl.59.5.662 12221154

[B9] Cehelyk E. K. Harvey D. Y. Grubb M. L. Jalel R. El-Sibai M. S. Markowitz C. E. (2019). Uncovering the association between fatigue and fatigability in multiple sclerosis using cognitive control. *Mult. Scler. Relat. Dis.* 27 269–275. 10.1016/j.msard.2018.10.112 30423531 PMC6442685

[B10] Compston A. Coles A. (2002). Multiple sclerosis. *Lancet* 372 1502–1517. 10.1016/S0140-6736(08)61620-7 18970977

[B11] Coyne J. C. van Sonderen E. (2012). No further research needed: abandoning the hospital and anxiety depression scale (HADS). *J. Psychosom. Res.* 72 173–174. 10.1016/j.jpsychores.2011.12.003 22325694

[B12] Du L. Zeng J. Liu H. Tang D. Meng H. Li Y. (2017). Fronto-limbic disconnection in depressed patients with suicidal ideation: a resting-state functional connectivity study. *J. Affect Disord.* 215 213–217. 10.1016/j.jad.2017.02.027 28340447

[B13] Eliasen A. Dalhoff K. P. Horwitz H. (2018). Neurological diseases and risk of suicide attempt: a case-control study. *J. Neurol.* 265 1303–1309. 10.1007/s00415-018-8837-4 29564603

[B14] Eshkoor S. A. Hamid T. A. Nudin S. S. H. Mun C. Y. (2014). The effects of social support, substance abuse and health care supports on life satisfaction in dementia. *Soc. Indic. Res.* 116 535–544. 10.1007/s11205-013-0304-0

[B15] Feinstein A. (2002). An examination of suicidal intent in patients with multiple sclerosis. *Neurology* 59 674–678. 10.1212/wnl.59.5.674 12221156

[B16] Fredrick S. S. Demaray M. K. Malecki C. K. Dorio N. B. (2018). Can social support buffer the association between depression and suicidal ideation in adolescent boys and girls? *Psychol. Sch.* 55 490–505. 10.1002/pits.22125

[B17] Gaskill A. Foley F. W. Kolzet J. Picone M. A. (2011). Suicidal thinking in multiple sclerosis. *Disabil. Rehabil.* 33 1528–1536. 10.3109/09638288.2010.533813 21091136

[B18] Gili-Planas M. Roca-Bennasar M. Ferrer-Perez V. Bernardo-Arroyo M. (2001). Suicidal ideation, psychiatric disorder, and medical illness in a community epidemiological study. *Suicide Life Threat.* 31 207–213. 10.1521/suli.31.2.207.21508 11459253

[B19] Goldberg D. P. Hillier V. F. (1979). A scaled version of the general health questionnaire. *Psychol. Med.* 9 139–145. 10.1017/s0033291700021644 424481

[B20] Goldberg D. P. Oldehinkel T. Ormel J. (1998). Why GHQ threshold varies from one place to another. *Psychol. Med.* 28 139–145. 9723146 10.1017/s0033291798006874

[B21] Hamilton T. K. Schweitzer R. D. (2000). The cost of being perfect: perfectionism and suicide ideation in university students. *Aust. N.Z. J. Psychiat.* 34 829–835. 10.1080/j.1440-1614.2000.00801.x 11037370

[B22] Henry A. Tourbah A. Camus G. Deschamps R. Mailhan L. Castex C. (2019). Anxietx and depression in patients with multiple sclerosis: the mediating effects of perceived social support. *Mult. Scler. Relat. Dis.* 27 46–51. 10.1016/j.msard.2018.09.039 30317070

[B23] Isernia S. Baglio F. d’Arma A. Groppo E. Marchetti A. Massaro D. (2019). Social mind and long-lasting disease: focus on affective and cognitive theory of mind in multiple sclerosis. *Front. Psychol.* 10:218. 10.3389/fpsyg.2019.00218 30792684 PMC6374311

[B24] Joshi P. Song H. B. Lee S. A. (2017). Association of chronic disease prevalence and quality of life with suicide-related ideation and suicide attempt among Korean adults. *Indian J. Psychiat.* 59:352. 10.4103/psychiatry.IndianJPsychiatry_282_16 29085096 PMC5659087

[B25] Kawabe K. Horiuchi F. Ochi M. Oka Y. Ueno S.-I. (2016). Suicidal ideation in adolescents and their caregivers: a cross sectional survey in Japan. *BMC Psychiatry* 16:231. 10.1186/s12888-016-0934-2 27400745 PMC4940687

[B26] Kim J. H. Park E. C. Cho W. H. Park J. Y. Choi W. J. Chang H. S. (2013). Association between total sleep duration and suicidal ideation among the Korean general adult population. *Sleep* 36 1563–1572. 10.5665/sleep.3058 24082316 PMC3773206

[B27] Kruithof W. J. van Mierlo M. L. Visser-Meily J. M. A. van Heugten C. M. Post M. W. M. (2013). Associations between social support and stroke survivors’ health-related quality of life – a systematic review. *Patient Educ. Couns.* 93 169–176. 10.1016/j.pec.2013.06.003 23870177

[B28] Kurtzke J. F. (1983). Rating neurologic impairment in multiple sclerosis – an expanded disability status scale (EDSS). *Neurology* 33 1444–1452. 6685237 10.1212/wnl.33.11.1444

[B29] Kye S. Y. Park K. (2017). Suicidal ideation and suicidal attempts among adults with chronic diseases: a cross-sectional study. *Compr. Psychiat.* 73 160–167. 10.1016/j.comppsych.2016.12.001 27992846

[B30] Lewis V. M. Williams K. KoKo C. Woolmore J. Jones C. Powell T. (2017). Disability, depression and suicide ideation in people with multiple sclerosis. *J. Affect. Disord.* 208 662–669. 10.1016/j.jad.2016.08.038 27866709

[B31] Lublin F. D. Reingold C. S. Cohen J. A. Cutter G. R. Sørensen P. S. Thompson A. J. (2014). Defining the clinical course of multiple sclerosis: the 2013 revisions. *Neurology* 83 278–286. 10.1212/WNL.0000000000000560 24871874 PMC4117366

[B32] Mellentin A. Stenager E. N. Stenager E. (2018). Preventing suicidal behavior in patients with multiple sclerosis: a scoping review. *Expert. Rev. Neurother.* 18 945–952. 10.1080/14737175.2018.1549990 30451039

[B33] Mileic S. Toncev G. Jevdjic J. Jovanovic B. Canovic D. (2011). Fatigue and depression in multiple sclerosis: correlation with quality of life. *Arch. Biol. Sci.* 63 617–622. 10.2298/abs1103617m

[B34] Newland P. Lorenz R. A. Smith J. M. Dean E. Newland J. Cavazos P. (2019). The relationship among multiple sclerosis-related symptoms, sleep quality, and sleep hygiene behaviors. *J. Neurosci. Nurs.* 51 37–42. 10.1097/JNN.0000000000000409 30489419

[B35] Nock M. K. Borges G. Bromet E. J. Alonso J. Angermeyer M. Beautrais A. (2008). Cross-National prevalence and risk facotrs for suicidal ideation, plans and attempts. *Br. J. Psychiat.* 192 98–105. 10.1192/bjp.bp.107.040113 PMC2259024 18245022

[B36] Østbye T. Yarnall K. S. H. Krause K. M. Pollak K. I. Gradison M. Michener J. L. (2005). Is there time for management of patients with chronic diseases in primary care? *Ann. Fam. Med.* 3 209–214. 10.1370/afm.310 15928223 PMC1466884

[B37] Polman C. H. Reingold S. C. Banwell B. Clanet M. Cohen J. A. Filippi M. (2011). Diagnostic criteria for multiple sclerosis: 2010 revisions to the McDonald criteria. *Ann. Neurol.* 69 292–302. 10.1002/ana.22366 21387374 PMC3084507

[B38] Rickards H. (2006). Depression in neurological disorders: an update. *Curr. Opin. Psychiatr.* 19 294–298. 10.1097/01.yco.0000218601.17722.5b 16612216

[B39] Rückert-Eheberg I. M. Lukaschek K. Brenk-Franz K. Strauß B. Gensichen J. (2019). Association of adult attachment and suicidal ideation in primary care patients with multiple chronic conditions. *J. Affect Disord.* 246 121–125. 10.1016/j.jad.2018.12.029 30580197

[B40] Sengul M. C. B. Kaya V. Sen C. A. Kaya K. (2014). Association between suicidal ideation and behavior, and depression, anxiety, and perceived social support in cancer patients. *Med. Sci. Monit.* 20 329–336. 10.12659/MSM.889989 24584172 PMC3945999

[B41] Smets E. M. A. Garssen B. Bonke B. De Haes J. C. (1995). The Multidimensional Fatigue Inventory (MFI) psychometric qualities of an instrument to assess fatigue. *J. Psychosom. Res.* 39 315–325. 10.1016/0022-3999(94)00125-o 7636775

[B42] Tauil C. B. Grippe T. C. Dias R. M. Dias-Carneiro R. P. C. Carneiro N. M. Aguilar A. C. R. (2018). Suicidal ideation, anxiety, and depression in patients with multiple sclerosis. *Arq. Neuro. Psiquiatr.* 76 296–301. 10.1590/0004-282X20180036 29898075

[B43] Vitkova M. Gdovinova Z. Rosenberger J. Szilasiova J. Mikula P. Stewart R. E. (2018). Is poor sleep quality associated with greater disability in patients with multiple sclerosis? *Behav. Sleep Med.* 16 106–116. 10.1080/15402002.2016.1173555 27191379

[B44] Watson D. Goldney R. Fisher L. Merritt M. (2001). The measurement of suicidal ideation. *Crisis* 22 12–14. 10.1027//0227-5910.22.1.12 11548814

[B45] Wilcon L. Whitehead L. Burrell B. (2011). Learning to live well with chronic fatigue: the personal perspective. *J. Adv. Nurs.* 67 2161–2169. 10.1111/j.1365-2648.2011.05666.x 21711464

[B46] Zigmond A. S. Snaith R. P. (1983). The hospital anxiety and depression scale. *Acta Psychiat. Scand.* 67 361–370. 6880820 10.1111/j.1600-0447.1983.tb09716.x

[B47] Zimet G. D. Dahlem N. W. Zimet S. G. Farley G. K. (1988). The multidimensional scale of perceived social support. *J. Pers. Assess.* 52 30–41. 10.1080/00223891.1990.9674095 2280326

