# Supplementary material for: Fatigue and Suicidal Ideation in People With Multiple Sclerosis: The Role of Social Support
Source: Front Psychol. 2020 Mar 18;11:504. doi: 10.3389/fpsyg.2020.00504 (PMC7093596; doi:10.3389/fpsyg.2020.00504)
Supplement: Supplementary file 1 [file Table_1.docx]

**Table 1: Description of the study population**

| **Variables** | **N (%)** | **Mean** | **Range** |
| --- | --- | --- | --- |
| **Age** | 156 | 39.95±9.97 | 18-61 |
| **Gender** |  |  |  |
| Male | 39 (25%) |  |  |
| Female | 117 (75%) |  |  |
| **Education** |  |  |  |
| Elementary | 6 (3.8%) |  |  |
| Secondary | 113 (72.4%) |  |  |
| Tertiary | 37 (23.8%) |  |  |
| **Disease duration (years)** | 151 | 7.40±5.48 | 1-28 |
| **EDSS** | 141 | 3.10±1.37 | 1-8 |
| **MS course** |  |  |  |
| CIS | 16 (11.5%) |  |  |
| RRMS | 97 (69.8%) |  |  |
| SPMS | 26 (18.7%) |  |  |
| **PSQI** | 155 | 5.77±3.54 | 0-16 |
| **MFI_GF** | 156 | 13.94±4.43 | 4-20 |
| **MFI_PF** | 156 | 13.37±4.86 | 4-20 |
| **MFI_RA** | 156 | 10.89±4.75 | 4-20 |
| **MFI_RM** | 156 | 8.52±3.64 | 4-20 |
| **MFI_MF** | 156 | 10.37±4.47 | 4-20 |
| **MSPSS** | 156 | 67.10±13.56 | 12-84 |
| **GHQ_SI** | 156 | 5.82±2.59 | 4-14 |
| **HADS depression** | 156 | 5.73±4.17 | 0-19 |

*Missing data: Disease duration (3.2%); EDSS (9.6%); PSQI (0.6%); MS course (10.8%). EDSS-Expanded Disability Status Scale; CIS-Clinically Isolated Syndrome; RRMS-Relapsing Remitting Multiple Sclerosis; SPMS-Secondary Progressive Multiple Sclerosis; PSQI-The Pittsburgh Sleep Quality Index; MFI_GF-General Fatigue; MFI_PF-Physical fatigue; MFI_RA-Reduced Activity; MFI_RM-Reduced Motivation; MFI_MF-Mental Fatigue; MSPSS-Multidimensional Scale of Perceived Social Support; GHQ_SI- Suicidal Ideation*
