# Supplementary material for: Fatigue and Suicidal Ideation in People With Multiple Sclerosis: The Role of Social Support
Source: Front Psychol. 2020 Mar 18;11:504. doi: 10.3389/fpsyg.2020.00504 (PMC7093596; doi:10.3389/fpsyg.2020.00504)
Supplement: Supplementary file 2 [file Table_2.docx]

**Table 2: Correlations between Sleep quality, types of fatigue, social support, EDSS and SI**

|  | Sleep quality | General fatigue | Physical fatigue | Reduced activity | Reduced motivation | Mental fatigue | Social support | EDSS | Suicidal ideation |
| --- | --- | --- | --- | --- | --- | --- | --- | --- | --- |
| Sleep quality |  |  |  |  |  |  |  |  |  |
| General fatigue | **0.46*** |  |  |  |  |  |  |  |  |
| Physical fatigue | **0.39*** | **0.80*** |  |  |  |  |  |  |  |
| Reduced activity | **0.39*** | **0.67*** | **0.79*** |  |  |  |  |  |  |
| Reduced motivation | **0.54*** | **0.58*** | **0.58*** | **0.66*** |  |  |  |  |  |
| Mental fatigue | **0.48*** | **0.57*** | **0.43*** | **0.46*** | **0.63*** |  |  |  |  |
| Social support | **-0.36*** | **-0.23*** | **-0.24*** | **-0.21*** | **-0.30*** | **-0.35*** |  |  |  |
| EDSS | 0.04 | **0.29*** | **0.41*** | **0.42*** | 0.10 | 0.02 | -0.03 |  |  |
| Suicidal ideation | **0.44*** | **0.32*** | **0.25*** | **0.27*** | **0.43*** | **0.45*** | **-0.34*** | -0.06 |  |
| HADS depression | **0.55*** | **0.60*** | **0.55*** | **0.64*** | **0.73*** | **0.67*** | **-0.43*** | **0.20*** | **0.55*** |

*EDSS-Expanded Disability Status Scale; *bold values: p<0.05*
