# Supplementary material for: Fatigue and Suicidal Ideation in People With Multiple Sclerosis: The Role of Social Support
Source: Front Psychol. 2020 Mar 18;11:504. doi: 10.3389/fpsyg.2020.00504 (PMC7093596; doi:10.3389/fpsyg.2020.00504)
Supplement: Supplementary file 3 [file Table_3.docx]

**Table 3: Linear regression analyses: Suicidal ideation regressed on demographic variables, EDSS, disease duration, sleep quality, social support and 5 types of fatigue**

|  | **Variables** | **Beta** | **F** | **Adjusted R^2^** |
| --- | --- | --- | --- | --- |
| **Model 1** | Age | -0.12 | 7.924 | 0.260 |
|  | Gender | -0.01 |  |  |
|  | EDSS | -0.01 |  |  |
|  | Disease duration | -0.14 |  |  |
|  | Sleep quality | **0.27*** |  |  |
|  | General fatigue | **0.20*** |  |  |
|  | Social support | **-0.25*** |  |  |
| **Model 2** | Age | -0.12 | 7.329 | 0.243 |
|  | Gender | -0.00 |  |  |
|  | EDSS | -0.02 |  |  |
|  | Disease duration | -0.14 |  |  |
|  | Sleep quality | **0.31*** |  |  |
|  | Physical fatigue | 0.14 |  |  |
|  | Social support | **-0.25*** |  |  |
| **Model 3** | Age | -0.13 | 8.083 | 0.264 |
|  | Gender | -0.03 |  |  |
|  | EDSS | -0.05 |  |  |
|  | Disease duration | -0.12 |  |  |
|  | Sleep quality | **0.28*** |  |  |
|  | Reduced activity | **0.23*** |  |  |
|  | Social support | **-0.23*** |  |  |
| **Model 4** | Age | -0.16 | 8.837 | 0.284 |
|  | Gender | -0.03 |  |  |
|  | EDSS | 0.03 |  |  |
|  | Disease duration | -0.13 |  |  |
|  | Sleep quality | **0.22*** |  |  |
|  | Reduced motivation | **0.28*** |  |  |
|  | Social support | **-0.22*** |  |  |
| **Model 5** | Age | -0.10 | 9.342 | 0.297 |
|  | Gender | -0.02 |  |  |
|  | EDSS | 0.03 |  |  |
|  | Disease duration | -0.16 |  |  |
|  | Sleep quality | **0.24*** |  |  |
|  | Mental fatigue | **0.29*** |  |  |
|  | Social support | **-0.21*** |  |  |

*EDSS-Expanded Disability Status Scale; *bold values: p<0.05*
